# Susceptibility of Children to Sapovirus Infections, Nicaragua, 2005–2006

**DOI:** 10.3201/eid1811.111581

**Published:** 2012-11

**Authors:** Filemón Bucardo, Beatrice Carlsson, Johan Nordgren, Göran Larson, Patricia Blandon, Samuel Vilchez, Lennart Svensson

**Affiliations:** University of León, León, Nicaragua (F. Bucardo, P. Blandon, S. Vilchez);; Linköping University, Linköping, Sweden (F. Bucardo, B. Carlsson, J. Nordgren, L. Svensson);; and University of Gothenburg, Göteborg, Sweden (G. Larson)

**Keywords:** sapovirus, disease suscceptibility, fucosyltransferase 2 gene, viruses, children, Nicaragua

## Abstract

We describe the genetic diversity of sapovirus (SaV) in children in Nicaragua and investigate the role of host genetic factors and susceptibility to SaV infections. Our results indicate that neither ABO blood group, Lewis phenotype, nor secretor status affects susceptibility to SaV infection in Nicaragua.

Human sapovirus (SaV), family *Caliciviridae*, is a causative agent of gastroenteritis in children and adults. Symptoms of SaV infection seem to be milder than symptoms of rotavirus and norovirus (NoV) infections; thus, SaV infection prevalence is higher in nonhospitalized children than in hospitalized children.

Some calciviruses bind to histo-blood group antigens (HBGA) expressed on cells in the gastrointestinal tract (*1*). Human NoV strains demonstrate strain-dependent binding patterns to HBGAs (*1*,*2*). ABO blood groups and Lewis phenotype also play a role in NoV infections, either as ligands or as restriction factors (*2*). Moreover, a non-sense mutation in the fucosyltransferase gene 2 (*FUT2*), which gives rise to the nonsecretor phenotype, has been found to provide almost complete protection from experimental and natural NoV infection (*2*,*3*).

Although human NoV susceptibility is highly associated with secretor status, and thus with mutations in *FUT2* (*4*), no information is yet available on whether host genetic factors determine susceptibility to SaV. We describe here the genetic diversity of SaV in a Central American population of hospitalized and nonhospitalized children and investigate the role of host genetic factors and susceptibility to SaV infections.

## The Study

A total of 292 children ≤5 years of age (205 symptomatic and 87 asymptomatic) were randomly selected among 694 symptomatic and 158 asymptomatic children who participated in a community- and hospital-based study of sporadic acute diarrhea in León, Nicaragua, during 2005–2006. In brief, clinical cases were evaluated according to the World Health Organization strategy for diarrhea management (*5*), and fecal, blood, and saliva samples were collected from each child.

Viral RNA was extracted from stool suspensions by use of a QIAmp Viral RNA Mini Kit (QIAGEN, Hilden, Germany), according to manufacturer’s instructions. RNA underwent reverse transcription (RT) to produce cDNA by using 50 pmol of random hexadeoxynucleotides (pd[N]6) (Amersham Biosciences, Chalfont St. Giles, United Kingdom) and RT-PCR beads (Amersham Biosciences), according to manufacturer´s instructions.

The RT products were amplified by SaV PCR using forward JV33 and reverse SR80 primers (*6*,*7*). This amplification was followed by an outer PCR with a primer pool of 2 forward (SV-F13 and SV-F14) and 2 reverse (SV-R13 and SV-R14) primers (*8*). Nested PCR was performed under identical conditions to the outer PCR with a primer pool consisting of universal forward primers (SV-F13 and SV-F14) and genogroup-specific reverse primers (SV-G1-R, SV-G2-R, SV-G4-R, and SV-G5-R). Primers for genogroup 3 were not available.

The N terminal and shell region of the SaV genome were then sequenced by using DYEnamic dye terminator kit (GE Healthcare, Little Chalfont, United Kingdom) (*9*). All samples included in the study were also screened for the presence of co-infection with the most common enteric pathogens (*5*). Any samples showing co-infection were then removed from further analysis.

Hemagglutination tests were performed to define blood types. The ABO blood group and Lewis phenotype in saliva were determined for persons found to be secretors as described (*10*).

A single nucleotide polymorphism at position 428 in the *FUT2* gene was investigated by pyrosequencing (*3*,*11*). Each person was classified as a homozygous secretor (SeSe), a heterozygous secretor (Sese^428^), or a nonsecretor (se^428^se^428^).

SaV-positive specimens were compared with specimens from a control group of children who tested negative for SaV, which was matched in terms of sex, age group, clinical status, and HBGAs using 2-tailed significance with χ^2^ or Fisher exact tests. GraphPad (GraphPad Software, Inc., La Jolla, CA, USA) was used for statistical analysis.

Of 292 samples analyzed, 33 (25 from symptomatic and 8 from asymptomatically infected children) (11%) were SaV positive. This study found that diarrhea is as common in SaV-infected children as it is in NoV-infected children in Nicaragua (*5*), and 11% of SaV-infected children were hospitalized (Table 1). Why the prevalence of SaV infections in Nicaraguan children is comparable to the high rate of NoV infections in these children is unknown and can only be speculated upon. Two likely key factors are poor sanitary conditions, which enables the virus to spread easily between susceptible persons, and malnourishment, which makes these children more susceptible to infection. Because we used conventional PCR with widely used JV33 and SR80 primers (*6*), the method of choice should not have created a bias.

**Table 1:** Epidemiologic profile of SaV infections, León, Nicaragua, 2005–2006*

Characteristic	Symptomatic, n = 205				Asymptomatic, n = 87		
	Total	No. (%) SaV strains	OR		Total	No. (%) SaV strains	OR
Setting							
Community	160	20 (12)	0.9		87	8 (9)	
Hospital	45	5 (11)			0		
Both	205	25 (12)			87	8 (9)	
Patient sex							
M	125	14 (11)	0.8		43	6 (14)	3.405†
F	80	11 (14)			44	2 (5)	
Patient age, mo							
≤6	36	5 (14)	1.2		11	2 (18)	2.6‡
7–12	57	12 (21)	2.8§		15	2 (13)	1.7
13–24	67	7 (10)	0.8		48	4 (8)	0.8
25– 60	45	1 (2)	0.1		13	0 (0)	NA

*SaV, sapovirus; OR, odds ratio; NA, not applicable. †Fisher test, 95%, CI, p = 0.15. ‡Fisher test, 95%, CI, p = 0.26. §Fisher test, 95%, CI, p = 0.02.

Results of a previous seroepidemiologic study of SaV infections suggested that SaV infections are frequent in children during the first 2 years of life because >90% of these children had antibodies against SaV (*12*). Our study supports this proposal because most SaV infections, either symptomatic or asymptomatic, occurred in children <2 years of age (Table 1).

**Figure 1:**
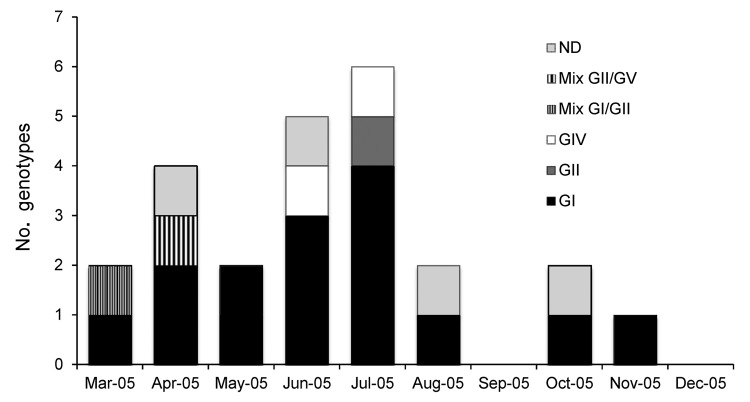
Distribution of sapovirus (SaV) genotypes in children ≤5 years of age from Nicaragua, March–December 2005. A total of 16 (64%) children were infected; 4 children (16%) were infected with genogroup II (GII), and 3 (12%) were infected with GIV. SaV infections were most frequently diagnosed during June–July 2005, in the rainy period. ND, not determined

SaV infections were most frequently detected during June–July 2005, in the rainy period (Figure 1), and were only found during March–November. The most common symptoms in SaV-positive children with diarrhea were loss of appetite (52%), vomiting (36%), fever (32%), and abdominal distension (32%).

**Figure 2:**
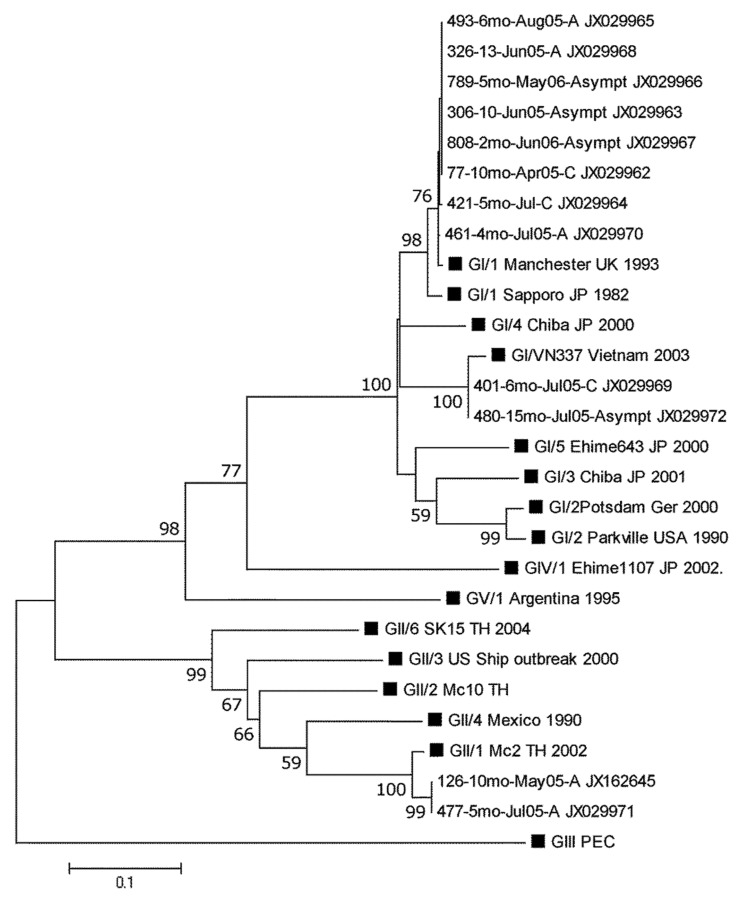
Phylogenetic analysis of the partial N terminal capsid gene (339 bp) of SaV strains identified in a pediatric population in Leon, Nicaragua, March 2005–September 2006. The tree was constructed on the basis of the Kimura 2-parameter and neighbor-joining methods with MEGA5 software (www.megasoftware.net). Bootstrap values are shown at the branch nodes (values <50% are not shown). The black squares represent SaV reference strains GI–GV. For Nicaraguan strains, the number of the strain is given, followed by age in months, month and year of sample collection, and clinical status. A, mild; C, severe; Asympt, asymptomatic. Scale bar indicates nucleotide substitutions per site.

To investigate whether certain SaV genogroups (GI, GII, GIV, or GV) were associated with clinical severity, we examined 25 SaV-positive symptomatic and asymptomatic children. All human SaV genogroups were found in study participants as observed (*13*). Notably, SaV GI was the dominant (64%) genogroup in Nicaragua, and infection with this genogroup could lead to asymptomatic disease as well as severe disease requiring hospitalization. This finding suggests that factors contributing to severity of SaV disease in Nicaragua are not only related to viral properties, but also to host factors and the surrounding environment. Co-infections with GI:GII and GI:GV were found in 2 symptomatic children, respectively. No association between genogroup and clinical status was observed; GI, GII, and GIV viruses infected symptomatic and asymptomatic children at similar rates (data not shown). Nucleotide sequencing of 12 SaV-positive samples revealed 8 additional strains related to the SaV Manchester strain, isolated in the United Kingdom in 1993, than to the SaV strain isolated in Japan in 1982, both representing genotype 1 within SaV GI (GI/1) (Figure 2).

We have previously investigated whether HBGA were susceptibility markers for NoV infections in children in Nicaragua and observed that secretors of blood type O were highly susceptible to infections with different NoV genotypes (*14*). No nonsecretors who were carrying the G428A *FUT2* mutation were infected with NoV (*9*,*14*). To investigate whether HBGAs were associated with SaV susceptibility similar to NoV susceptibility (*2*,*3*,*11*), we examined 22 of 33 SaV-infected children who were either symptomatic (n = 18) or asymptomatic (n = 4) in relation to ABO blood types, Lewis phenotypes, and secretor genotype. Notably, we did not find any significant association between ABO blood groups, Lewis phenotypes, or secretor genotype and susceptibility to SaV infection (Table 2). Children who were secretors (SeSe and Sese^428^) and nonsecretors (se^428^se^428^ and Le^a+b-^) were susceptible to SaV infections. Our data suggest that SaV can infect secretors and nonsecretors, those who are Lewis phenotype positive as well as Lewis phenotype negative, and persons of all ABO blood groups. However, it must be noted that these data represent susceptibility to symptomatic GI SaV infection. To our knowledge, only 1 report in the literature has explored binding properties of recombinant SaV GI/1 (Mc114) and GV/1 (NK24) to HBGAs and revealed that the recombinant SaV strains investigated showed no specific binding activity to HBGAs from or to synthetic carbohydrates (*15*).

**Table 2:** Distribution of ABO blood groups, Lewis antigens, and *FUT2* 428 genotypes among SaV- infected children and healthy controls, León, Nicaragua, 2005–2006*

Variable	No. (%) SaV infected	No. (%) controls†	OR (95% CI)	p value‡
Blood group				
O	16 (73)	87 (66)	1.3 (0.49–3.7)	0.63
A	2 (9)	26 (20)	0.4 (0.09–1.8)	0.37
B	3 (13.5)	13 (10)	1.4 (0.37–5.5)	0.82
AB	1 (4.5)	5 (4)	1.2 (0.13–11)	1.00
Lewis phenotype				
Le^a-b+^	17 (77.5)	93 (71)	1.3 (0.46–3.91)	1.00
Le^a+b-^	2 (9)	5 (4)	2.5 (0.45–13.7)	0.26
Le^a-b-^	3 (13.5)	32 (25)	0.48 (0.13−1.7)	0.41
*FUT2* SNP (428G→A)				
SeSe	13 (59)	69 (53)	1.3 (0.52–3.2)	0.64
Sese^428^	7 (32)	58 (44)	0.59 (0.22–1.5)	0.35
se^428^se^428^	2 (9)	4 (3)	3.2 (0.55–18.00)	0.2
Secretor status				
Secretor	20 (91)	123 (94)	0.65 (0.13–3.3)	0.63
Nonsecretor	2 (9)	8 (6)	1.5 (0.3–7.8)	0.63

*SaV, sapovirus; OR, odds ratio; *FUT2*, fucosyltransferase gene 2; SNP, single nucleotide polymorphism; SeSe, homozygous wild type at *FUT2* 428; Sese_428_, heterozygous carrier of *FUT2* 428 non-sense mutation; se_428_se_428_, homozygous carrier of the non-sense mutation at nt 428 in the *FUT2* gene †From Bucardo et al. (*14*). ‡Fischer exact test.

## Conclusions

Our results demonstrate that SaV, similar to NoV, frequently causes acute gastroenteritis in children in Nicaragua (*5*). However, in contrast to the case with NoV, neither the 428A nonsecretor mutation, nor any of the ABO blood groups or Lewis phenotypes protected children against SaV infection.
